# What can be found in the spam folder? a self-study from junior researchers in psychiatry

**DOI:** 10.1192/bjo.2021.671

**Published:** 2021-06-18

**Authors:** Nikhil Gauri Shankar, Jashan Selvakumar, Jiann Lin Loo, Mary Honey Ohn, Sze Hung Chua, Asha Dhandapani, Manjula Simiyon, Jawad Raja

**Affiliations:** 1 Ysbyty Maelor Wrecsam, Betsi Cadwaladr University Health Board; 2 St George's University of London; 3 University Hospital Lewisham; 4 Hospital Bahagia Ulu Kinta

## Abstract

**Aims:**

Thriving on the pressure of “publish or perish” experienced by academicians, the industry of predatory publishers with dubious quality has mushroomed and gained their notoriety. The battle of uncovering predatory publishers, including Beall's list, has proven to be tough given the huge monetary gain generated by the predatory publishers. It may be difficult for an inexperienced junior researcher to identify those predatory publishers’ soliciting emails, which may disguise as a reputable journal's article-commissioning process. To date, there is a limited systematic approach to identify such emails. Hence, this research is aimed to describe the common features of soliciting emails from publishers which appeared to be predatory.

**Method:**

This self-study involved reviewing the content of emails in the spam folder of authors, a team of junior researchers in psychiatry, for a month. Emails included in this study were soliciting emails relevant to publications and the following were reviewed: types of solicitation, sentences used, strategies used, and information available in the public domain of their webpages. Informative types of emails were excluded.

**Result:**

The solicitation could include: 1) request for a manuscript to be published a journal article, 2) request for a thesis to be published as a book, 3) request to write for a book chapter, 4) invitation to be an editorial member or a reviewer with the offer of free publishing, 5) invitation to be a speaker for a conference, and 6) proofreading services. The publisher may cite a published article of the author from another journal, which was the source where they identified the author's email. Common strategies used for solicitation included: 1) promising a fast-tracked and guaranteed publication, 2) using compliments that appeared to be inappropriate, 3) repetitive emails, and 4) using argumentum ad passiones to induce guilt. The common features of the webpages of those publishers included: 1) open access publishing as the only option, 2) extensive list of indexing services excluding well-established indexing agencies, and 3) the publisher has a huge collection of journals in different disciplines.

**Conclusion:**

It is hoped that these findings will help junior researchers in psychiatry to stay vigilant to avoid falling into the trap of predatory publishers, which may result in financial loss and loss of work to plagiarism. Total eradication of those predatory soliciting emails is unlikely despite the advancement of spam filtering technology, which necessitates a more united effort from different stakeholders to come out with a probable solution.

